# Influence of *MC1R* Gene Variants on Coat Color of Indicine Cattle Breeds

**DOI:** 10.1002/age.70113

**Published:** 2026-05-02

**Authors:** Silel V. S. A. Maciel, Ingrid P. P. Oliveira, Beatriz B. Senes, Carolaine J. S. Santana, Jackeline S. Alves, Edilane A. da Silva, Fernando O. Franco, Bárbara C. F. da Silva, Fernando C. Cairo, Frederico C. Cairo, Aníbal E. Vercesi‐Filho, Raphael B. Costa, Gregório M. F. de Camargo

**Affiliations:** ^1^ Escola de Medicina Veterinária e Zootecnia da Universidade Federal da Bahia (UFBA) Salvador Bahia Brazil; ^2^ Empresa de Pesquisa Agropecuária de Minhas Gerais (Epamig) Belo Horizonte Minas Gerais Brazil; ^3^ Universidade Estadual do Sudoeste da Bahia (UESB) Itapetinga Bahia Brazil; ^4^ Instituto de Zootecnia Nova Odessa São Paulo Brazil

**Keywords:** black coat color, *Bos taurus indicus*, dark skin, depigmentation, indel, SNP, white markings

## Abstract

Indicine cattle display a broad range of coat colors, representing a relevant and breed‐defining trait required for registration in breeder association. Among the genes involved in pigmentation, *MC1R* stands out for modulating the synthesis of melanin‐related proteins and remains poorly explored in indicine cattle breeds. Therefore, we aimed to perform fine mapping of the *MC1R* gene and to evaluate the association between its polymorphisms and coat color as well as skin pigmentation in seven zebu cattle breeds. A total of 295 individuals belonging to Gir, Nelore, Sindi, Indubrasil, Tabapuã, Guzerá, and Brahman cattle breeds were genotyped through PCR‐sequencing, followed by estimates of genotypic and allelic frequencies, linkage disequilibrium, and association with phenotypes using the Kruskal–Wallis test. We identified nine polymorphisms: six non‐synonymous SNPs, one coding indel, one synonymous SNP, and one 3′ downstream SNP. We report, for the first time, the SNP c.274G>A (p.Val92Met), here designated *E* ^*Z1*^, and SNP c.871G>A (p.Ala291Thr) associated with the dark skin phenotype in Gir cattle, here designated *E* ^*Z2*^. Additionally, the indel c.310del was associated with depigmentation in Sindi, Guzerá, and Gir cattle breeds. The variants described herein represent potential molecular markers to support selection strategies targeting desired coat and pigmentation phenotypes.

## 1 Introduction

Indicine cattle originate from tropical environments characterized by high temperatures, strong solar incidence, and limited availability of high‐quality forage, which has favored the evolution of physiological traits related to adaptation (Nayak et al. 2024). Coat and skin pigmentation directly contribute to thermoregulation (Maibam et al. 2018) and also influence ectoparasite adhesion (Horner and Gomes 1990; Saueressig 1992; Oliveira et al. 2013; Cardoso et al. 2014; López‐Herrera and Briceño‐Arguedas 2014).

In addition to adaptive importance, coat and skin color constitute a major characteristic for breed qualification, determining whether an animal is eligible for registration at breeder association. In the Brazilian Zebu Cattle Breeders Association (ABCZ in Portuguese acronym), responsible for registering all zebu breeds in Brazil, specific coat color phenotypes and mucosal pigmentation are mandatory for registration (ABCZ 2025). Animals that deviate from established patterns—such as depigmented individuals—are not eligible for registration (Appendix S1–S1.4), and thus exhibit reduced commercial value. Furthermore, breeds presenting multiple coat color variations, such as Gir (Appendix S1–S1.2.2), often show breeder preference toward specific patterns.

Coat color is a polygenic trait (Cichorek et al. 2013), with 688 pigmentation‐associated genes currently reported across multiple species (Baxter et al. 2022). Among these is *MC1R*, which encodes a membrane receptor that is coupled to a G protein (GPCR) located in the melanocyte membrane, with a strong influence on the synthesis of pigment type. The function of this receptor is modulated by antagonistic interactions between the alpha melanocyte‐stimulating hormone (α‐MSH) and the Agouti signaling protein (ASIP) (Ji and Tao 2022; Kleinau et al. 2024).

Variations in the *MC1R* gene determine coat color through gain‐of‐function or loss‐of‐function mechanisms. In the presence of the dominant allele (ED), a gain‐of‐function occurs where the receptor becomes constitutively active resulting in the production of eumelanin (black and brown pigments), thereby masking the action of the ASIP gene through epistasis. Conversely, when a loss‐of‐function mutation occurs (allele *e*), the receptor becomes inactive, leading to the production of pheomelanin (red and yellow pigments) independent of the presence of *ASIP* (Hearing and Tsukamoto 1991; Chen et al. 2017; Neves et al. 2017; Ji and Tao 2022). In cases involving the wild‐type allele (*E* ^+^), the receptor is functional, and there is antagonistic competition between the α‐MSH and Agouti proteins for the receptor, which may result in the production of either eumelanin or pheomelanin (Adalsteinsson et al. 1995; Royo et al. 2005; Han et al. 2011; Ji and Tao 2022). Numerous *MC1R* polymorphisms have been described, and together with *ASIP*, they shape much of the chromatic diversity observed in cattle (Hauser et al. 2022).

Furthermore, genes such as *PMEL* and *MLPH* influence coat color dilution, resulting in phenotypes such as gray and blue in breeds like Highland, Holstein, and Belgian Blue, while the *ASIP* gene influences coat darkening in specific body regions in Nelore and Brahman cattle (Schmutz and Dreger 2013; Dikmen et al. 2017; Laible et al. 2021; Trigo et al. 2021, 2023). Additionally, variants in the *COPA* gene were identified as causing the “Dominant Red” phenotype, mimicking the loss of function of *MC1R* by altering synthesis from eumelanin to pheomelanin (Dorshorst et al. 2015).

Regarding the occurrence of white markings, the main genes related to this phenotype are present in the *KIT signaling pathway* (including *KIT*, *KITLG*, and *MITF*), frequently involving noncoding regulatory variants. This predominance of the *KIT*/*MITF*/*PAX3* pathway in the formation of white spotting is widely supported by genome‐wide association and structural variant studies that highlight the crucial role of these genes and their regulators (such as LncRNAs) in melanoblast migration and survival during embryogenesis in diverse breeds, such as Reggiana, Simmental, Sahiwal, and Hereford (Fontanesi et al. 2010; Pausch et al. 2012; Durkin et al. 2012; Bovo et al. 2021; Illa et al. 2021; Jara et al. 2022). It has also been reported that epistatic interactions with the *MC1R* genotype modulate the distribution and appearance of white spotting in mammals (Kijas et al. 2001; Fontanesi et al. 2009; Maciel et al. 2020; Patterson Rosa et al. 2022). However, few studies have been conducted to understand the occurrence of markings in zebu cattle, requiring further research (Maciel et al. 2024).

Although *MC1R* has been extensively studied in *Bos taurus taurus* cattle (Klungland et al. 1995; Rouzaud et al. 2000; Kriegesmann et al. 2001; Graphodatskaya 2002; Mohanty et al. 2008; Guastella et al. 2011; Matsumoto et al. 2020; Hauser et al. 2022; He et al. 2022), available information in *Bos taurus indicus* and crossbred populations remains scarce, with reports limited to breeds such as Zhoushan and Wenling (Jiang et al. 2021), Karan Fries, Tharparkar and Sahiwal (Goud et al. 2019, 2021), and Guzerá (Santana et al. 2021). The genomic region harboring *MC1R* has also been proposed as a candidate locus for coat color regulation in Gir (Maciel et al. 2024), but much of the genetic basis underlying pigmentation in indicine cattle remains unresolved.

## 2 Material and Methods

### 2.1 Animals and Phenotypes

This study was approved by the Animal Ethics Committee of the Escola de Medicina Veterinária e Zootecnia of the Universidade Federal da Bahia (project number approval 29/2021). A total of 295 animals representing seven zebu breeds with distinct coat color profiles were evaluated: Gir (*n* = 119) (Table 1A), Nelore (*n* = 73), Sindi (*n* = 42), Indubrasil (*n* = 20), Tabapuã (*n* = 18), Guzerá (*n* = 14), and Brahman (*n* = 9) (Table 1B).

**TABLE 1:** (A) Distribution of animals by coat color in Gir cattle. (B) Distribution of animals by coat color in other indicine breeds.

	Coat color													Total of animals
	R	RCH	RC	CR	Y	YCH	YC	CY	CC	DSL	DSR	DSD	DM^a^	
**A. Breed**														
Gir	10	10	10	10	11	7	11	10	11	10	10	8	1	119

	R	W	G	SR	SBL	Y	B	BL	DM^a^	RO	—	—	—	
**B. Other breeds**														
Nelore	12	12	8	23	12	1	3	1	1	—	—	—	—	73
Sindi	39	—	—	—	—	—	—	—	3	—	—	—	—	42
Indubrasil	1	11	8	—	—	—	—	—	—	—	—	—	—	20
Tabapuã	—	17	1	—	—	—	—	—	—	—	—	—	—	18
Guzerá	—	—	10	—	—	—	—	—	1	3	—	—	—	14
Brahman	2	6	1	—	—	—	—	—	—	—	—	—	—	9

Abbreviations: B, blue; BL, black; CC, calico clear; CR, calico red; CY, calico yellow; DM, depigmented mucosa; DSD, dark skin, dark coat color; DSL, dark skin, light coat color; DSR, dark skin, red head; G, gray; R, red; RC, red calico; RCH, red choker; RO, roan; SBL, spotted black; SR, spotted red; W, white; Y, yellow; YC, yellow calico; YCH, yellow choker.

^a^ Depigmented animal (not registered).

Breed names and coat color categories (Portuguese–English), along with reference images (Appendix S1), and the frequency distribution of color patterns within each breed (Table 1) are provided. For Guzerá, 11 of the 14 individuals (10 gray and 1 depigmented) were sourced from Santana et al. (2021). Six animals in the dataset were depigmented and were not registered in ABCZ (Table 1, Appendix S1–S1.4).

Throughout the article, coat color nomenclature is presented in English. Breed names follow DAD‐IS/FAO recommendations (https://www.fao.org/dad‐is/browse‐by‐country‐and‐species/en/) and were presented in Portuguese.

### 2.2 DNA Extraction, PCR Amplification, and *MC1R* Sequencing

Genomic DNA was extracted from hair follicles using the NucleoSpin Tissue Kit following the manufacturer's protocol. DNA quality was assessed using NanoDrop spectrophotometry by measuring DNA concentration and A260/A280 ratio, which was considered acceptable at values between 1.8 and 2.0. DNA samples were diluted to a standard concentration of 100 ng/μL.

PCR amplification targeted the entire *MC1R* coding region using primer sets described by Hauser et al. (2022), under the cycling conditions established by the same authors. Reactions were performed in an Applied Biosystems SimpliAmp Thermal Cycler (ThermoFisher Scientific Inc., Waltham, MA, USA). Amplicons were visualized by 1.5% agarose gel electrophoresis, purified using 20% PEG, quantified, and sequenced bidirectionally using an Applied Biosystems 3500xL Genetic Analyzer. Sequence editing was carried out in BioEdit v7.0.9.0 (Hall 1999), and alignments were generated using the ClustalW algorithm (Hall 1999; Thompson et al. 2002) utilizing the *Bos taurus* *MC1R* sequence (NC_037345.1:14705093–14706843) as the reference. Identified variants were described according to HGVS nomenclature based on the *Bos taurus* *MC1R* RefSeq transcript NM_174108.2 and BTA18 from the ARS‐UCD2.0 assembly (NC_037345.1).

Subsequently, gametic phase inference (phasing) for haplotype reconstruction was performed using DnaSP v6.12.03 software (Rozas et al. 2017). The resolution of heterozygous positions into discrete haplotypes was conducted by applying the PHASE algorithm (Stephens et al. 2001) that assumes Hardy–Weinberg equilibrium and uses a coalescent based Bayesian method to infer haplotypes.

To evaluate the potential functional impacts of the identified mutations, the Ensembl Variant Effect Predictor (VEP) web tool (https://www.ensembl.org/Tools/VEP) was employed.

Finally, the representative sequences of each breed were deposited in GenBank under the accession numbers: PQ461102 (Gir), PQ461103 (Sindi), PQ461104 (Guzerá), PQ461105 (Tabapuã), PQ461106 (Brahman), PQ461107 (Nelore), and PQ461108 (Indubrasil).

### 2.3 Compilation of *MC1R* Sequence Variants and Structural Protein

All previously reported *MC1R* polymorphisms (ENSBTAG00000023731) were retrieved from Ensembl (version 115, 2025). Search parameters included genomic interval chr18:14705375–14706329, encompassing the *MC1R* coding region, allowing extraction of all known variants for comparison with results obtained in the present study.

To visualize the transmembrane topology of the Melanocortin 1 Receptor (MC1R), the PROTTER web‐based tool (Omasits et al. 2014) was utilized. The topological representation was constructed using the bovine MC1R amino acid sequence retrieved from the UniProtKB database (accession number P47798). This structural model was annotated to map the functional missense mutations identified in our study, alongside all previously reported functional variants with known phenotypic effects on bovine coat color.

### 2.4 Data Analysis

Linkage disequilibrium (LD) analysis among the identified *MC1R* variants and haplotype block visualization were performed using Haploview v4.2 software (Barrett et al. 2005). The degree of nonrandom association between alleles at different loci was estimated utilizing the normalized coefficient of linkage disequilibrium (*D*′) and the squared allele frequency correlation coefficient (*r* ^2^) described by Gabriel et al. (2002).

Statistical analyses were conducted in R version 4.4.1 (R Core Team 2024). Allelic and genotypic frequencies were estimated using the *genetics* package v1.3.8.1.3 (Warnes et al. 2021). To investigate genotype–phenotype associations, statistical tests were performed at the individual SNP level. For this purpose, phenotype‐based grouping scenarios were defined for coat color comparisons (Table 2), with particular emphasis on the Gir and Nelore breeds due to their broader coat color variation. Finally, Kruskal–Wallis tests were applied using the base *stats* package to detect significant SNP‐phenotype associations across these scenarios.

**TABLE 2:** Scenarios used for Kruskal–Wallis tests.

Scenario (*n*)	Phenotype groups/coat colors (*n*)
**Gir cattle**	
GS1 (119)	R (11) vs. RCH (10) vs. RC (10) vs. CR (10) vs. Y (11) vs. YCH (7) vs. YC (11) vs. CY (10) vs. DSL (10) vs. DSD (8) vs. DSR (10) vs. CC (11)
GS2 (80)	Solid (22) (*R* + Y) vs. spotted (58) (RCH + RC + CR + YCH + YC + CY)
GS3 (120)	P (119) vs. DM (1)
GS4 (119)	Dark skin (28) (DSL + DSD + DSR) vs. not dark skin (91) (*R* + RCH + RC + CR + Y + YCH + YC + CY + CC)
GS5 (80)	Red (41) (*R* + RCH + RC + CR) vs. yellow (39) (Y + YCH + YC + CY)
GS6 (39)	Solid (22) (*R* + Y) vs. choker (17) (RCH + YCH)
GS7 (43)	Solid (22) (*R* + Y) vs. calico (less spots) (21) (RC + YC)
GS8 (33)	Solid (22) (*R* + Y) vs. calico clear (11) (CC)
GS9 (50)	Solid (22) (*R* + Y) vs. dark skin (28) (DSL + DSD + DSR)
GS10 (42)	Solid (22) (*R* + Y) vs. calico (20) (more spots) (CR + CY)
GS11 (69)	R (11) vs. spotted (58) (RCH + RC + CR + YCH + YC + CY)
**Nelore cattle**	
NS1 (72)	Y (1) vs. W (12) vs. B (3) vs. G (8) vs. BL (1) vs. SBL (12) vs. R (12) vs. SR (23)
NS2 (72)	Black (13) (BL+ SBL) vs. not black (59) (Y + W + B + G + *R* + SR)
NS3 (72)	Red (35) (*R* + SR) vs. not red (37) (Y + W + B + G + BL + SBL)
NS4 (72)	Gray (8) (G) vs. not gray (64) (Y + W + B + BL + SBL + *R* + SR)
NS5 (72)	Blue (3) (B) vs. not blue (69) (Y + W + G + BL + SBL + *R* + SR)
NS6 (72)	Spotted (35) (SBL + SR) vs. solid (37) (Y + W + B + G + BL + *R*)
NS7 (73)	P (72) vs. DM (1)
NS8 (56)	Red (35) (*R* + SR) vs. not red (21) (Y + W + G), without black and blue coat color
**Sindi cattle**	
SS1 (42)	P (39) vs. DM (3)
**Indubrasil cattle**	
IS1 (20)	W (11) vs. G (8) vs. R (1)
**Tabapuã cattle**	
TS1 (18)	W (17) vs. G (1)
**Guzerá cattle**	
GUS1 (14)	G (10) vs. DM (1) vs. RO (3)
GUS2 (14)	“With breed qualification” (10) (G) vs. “Without breed qualification” (4) (DM + RO)
**Brahman cattle**	
BS1 (9)	W (6) vs. G (1) vs. R (2)
**All breeds**	
All1 (296)	P (291) vs. DM (5)

Abbreviations: B, blue; BL, black; CC, calico clear; CR, calico red; CY, calico yellow; DM, depigmented mucosa; DSD, dark skin, dark coat color; DSL, dark skin, light coat color; DSR, dark skin, red head; G, gray; *n*, number of animals; P, pigmented; R, red; RC, red calico; RCH, red choker; RO, roan; SBL, spotted black; SR, spotted red; vs., versus; W, white; Y, yellow; YC, yellow calico; YCH, yellow choker.

## 3 Results

Fine‐scale mapping of the *MC1R* coding region revealed nine polymorphisms: seven coding SNPs, one coding indel, plus one noncoding SNP located 3′ downstream (Table 3). Among the coding SNPs, six were missense mutations and the indel introduced a frameshift resulting in premature stop codon formation. Three population‐specific SNPs were detected—one in Nelore c.296 (NM_174108.2:c.296T>C) and two in Gir c.416 (NM_174108.2:c.416C>T), c.460 (NM_174108.2:c.460A>C) (Table 3). Allele and genotype frequencies were estimated for each breed and coat color category (Appendix S2).

**TABLE 3:** Genomic coordinates, functional impact, and breed distribution of *MC1R* variants identified in Zebu cattle.

Chromosome position	CDS position	Existing variation	cDNA position	Ref allele	Altered allele	Consequence	Protein position	Amino acids	Codons	Impact	SIFT	Breeds of occurrence in present work
NC_037345.1:g.14705649G>A	NM_174108.2:c.274G>A	—	729	G	A	Missense_variant	NP_776533.1:Val92Met	V/M	Gtg/Atg	Moderate	Tolerated (0.13)	Gir and Nelore
NC_037345.1:g.14705671T>C	NM_174108.2:c.296T>C	rs109688013	751	T	C	Missense_variant	NP_776533.1:Leu99Pro	L/P	cTg/cCg	Moderate	Deleterious (0.01)	Nelore
NC_037345.1:g.14705686Gdel	NM_174108.2:c.310del	rs110710422	765	G	—	Frameshift_variant	NP_776533.1:Gly104ValfsTer50	G/V	Ggt/gt	High	—	Brahman, Gir, Guzerá, Nelore, Sindi, and Tabapuã
NC_037345.1:g.14705791C>T	NM_174108.2:c.416C>T	rs524530585	871	C	T	Missense_variant	NP_776533.1:Ala139Val	A/V	gCt/gTt	Moderate	Deleterious (0)	Gir
NC_037345.1:g.14705835A>C	NM_174108.2:c.460A>C	rs5351585564	915	A	C	Missense_variant	NP_776533.1:Ser154Arg	S/R	Agt/Cgt	Moderate	Deleterious (0)	Gir
NC_037345.1:g.14705958C>T	NM_174108.2:c.583C>T	rs520119552	1038	C	T	Missense_variant	NP_776533.1:Leu195Phe	L/F	Ctc/Ttc	Moderate	Tolerated (1)	Brahman, Gir, Guzerá, Nelore, and Sindi
NC_037345.1:g.14706038T>C	NM_174108.2:c.663T>C	rs525311468	1118	T	C	Synonymous_variant	NP_776533.1:I221	I	atT/atC	Low	—	Brahman, Gir, Guzerá, Nelore, and Sindi
NC_037345.1:g.14706246G>A	NM_174108.2:c.871G>A	rs135181132	1326	G	A	Missense_variant	NP_776533.1:Ala291Thr	A/T	Gcc/Acc	Moderate	Deleterious (0.03)	Gir and Nelore
NC_037345.1:g.14706342C>T	NM_174108.2:c.967C>T	rs714330082	1422	C	T	3_prime_UTR_variant	—	—	—	Modifier	—	Brahman, Gir, Guzerá, Indubrasil, Nelore, and Tabapuã

Haplotype reconstruction was based only on the variants identified in the coding region of the *MC1R* gene, revealing the presence of 11 haplotypes distributed heterogeneously among the Zebu breeds evaluated (Table 4). Haplotype 4 (H4) and the H4/H4 diplotypic combination presented the highest frequencies in the overall population, suggesting that H4 represents the “Indicine consensus haplotype” form of the *MC1R* gene in the *Bos taurus indicus* . Association analyses were not done with diplotypes due to the extremely rare frequency of some of them (Appendix S3).

**TABLE 4:** Definition of the *MC1R* haplotypes identified in seven Zebu cattle breeds.

	c.274	c.296	c.310	c.416	c.460	c.583	c.663	c.871
Hap 1^a^	G	T	G	C	A	C	T	G
Hap 2	—	—	—	—	—	—	C	—
Hap 3	—	—	—	T	C	—	C	—
Hap 4	—	—	—	—	—	T	C	—
Hap 5	—	—	DEL	—	—	—	—	—
Hap 6	—	—	DEL	—	—	—	—	A
Hap 7	—	—	—	T	C	T	C	—
Hap 8	—	—	—	—	—	—	—	A
Hap 9	A	—	—	—	—	—	—	A
Hap 10	—	—	—	—	—	T	T	A
Hap 11	—	C	—	—	—	—	—	—

^a^ Hap 1 was based on the reference sequence NC_037345.1:14705093–14706843 (ARS‐UCD2.0).

The LD matrices were generated for within‐breed comparisons, revealing distinct patterns of genomic structuring among the evaluated breeds. Different numbers of haplotype blocks were generated: two in the Gir breed and one in the Nelore, Sindi, and Guzerá breeds (Appendix S4). For the Tabapuã and Brahman breeds, no haplotype block formation was detected, while for the Indubrasil breed, LD could not be calculated due to the absence of polymorphisms in the coding region. Overall, it was observed that the *D*‐prime (*D*′) value was equal to 1 in most comparisons, with the exception of some variant pairs in the Gir breed. These results demonstrate that, in most cases, the SNPs show no evidence of historical recombination events, being inherited together within their respective haplotype blocks.

Associations between SNPs and pigmentation phenotypes were evaluated via Kruskal–Wallis tests (Appendix S5) and the positions that showed significance in the scenarios can be seen in Table 5.

**TABLE 5:** Significant SNP‐phenotype association scenarios^a^.

Scenarios	Significative positions (*p* value)	Phenotype association
Gir		
GS1	871 (0.05)	All coat colors
GS2	583 (0.002); 663 (0.04)	Solid × spotted
GS3	310 (0.004); 416 (0.002); 460 (0.002); 583 (0.003)	Pigmented × depigmented
GS4	274 (0.01); 583 (2.51^−07^); 663 (1.26^−11^); 871 (2.22^−16^)	Dark skin × others
GS6	583 (0.05)	Solid × spots in dewlap
GS7	310 (0.04); 583 (0.04)	Solid × few spots
GS9	310 (0.05); 583 (0.02); 663 (0.0003); 871 (1.11^−07^)	Dark skin × solid
GS10	416 (0.04); 460 (0.04); 583 (0.01)	Solid × many spots
Nelore		
NS1	583 (0.03); 663 (0.03)	All coat colors
NS2	274 (3.36^−12^); 583 (6.96^−08^); 663 (6.96^−08^); 871 (4.97^−12^)	Black × not black
NS3	274 (0.001); 583 (9.65^−05^); 663 (9.65^−05^); 871 (0.002)	Red × not red
NS5	296 (6.02^−12^)	Blue × not blue
NS6	274 (0.03)	Red × not red (without black and blue)
Sindi		
SS1	310 (1.2^−06^)	Pigmented × depigmented
Guzerá		
GUS1	310 (0.002); 583 (0.004); 663 (0.004)	All coat colors
GUS2	310 (0.002); 583 (0.004); 663 (0.004)	With breed qualification × without breed qualification
All animals		
All1	310 (< 2.0^−16^)	Pigmented × depigmented

^a^ To check *p* values and detailed scenarios, see Appendix S5.

Due to the expressive statistical significance obtained for specific variants (Table 5), a survey of all previously reported polymorphisms for the *MC1R* gene was conducted with the aim of investigating known associations with bovine coat color. Of the 229 variants previously listed for *MC1R* in *Bos taurus* (Appendix S6), it was identified that 12 of them present proven functional effects on coat coloration in cattle (Table 6).

**TABLE 6:** Standard nomenclature, polymorphisms, and reported functional effects of bovine *MC1R* alleles.

Allele	Functional effect	Polymorphism	Amino acid change	References
e^V2^	Recessive red	NM_174108.2:c.263G>A	NP_776533.1:S88N	Hauser et al. (2022)
E^Z1^	Dominant black	NM_174108.2:c.274G>A	NP_776533.1:V92M	Present work
E^D^	Dominant black	NM_174108.2:c.296T>C	NP_776533.1:L99P	Klungland et al. (1995), Rouzaud et al. (2000), Kriegesmann et al. (2001), Graphodatskaya (2002), Mohanty et al. (2008), Guastella et al. (2011), Hanna et al. (2014), Goud et al. (2019), Matsumoto et al. (2020), Santana et al. (2021), He et al. (2022)
E^+^	Wild type	NM_174108.2:c.296T	NP_776533.1:L99	
e	Recessive red	NM_174108.2:c.310del	NP_776533.1:G104VfsTer50	
e^V1^	Recessive red	NM_174108.2:c.424C>T	NP_776533.1:R142C	Hauser et al. (2022)
E^d2^	—	NM_174108.2:c.651_662dup	NP_776533.1:220_223dup	Graphodatskaya (2002), Hauser et al. (2022)
E^d1^/E^2^	—	NM_174108.2:c.667 C>T	NP_776533.1:R223W	Graphodatskaya (2002), Guastella et al. (2011), Hauser et al. (2022)
E^1^	—	NM_174108.2:c.670_681dup	NP_776533.1:224_227dup	Rouzaud et al. (2000), Kriegesmann et al. (2001), Guastella et al. (2011)
p.T281N	Red Sahiwal	NM_174108.2:c.844C>A	NP_776533.1:T281N	Goud et al. (2021)
E^Z2^	Dominant darkening	NM_174108.2:c.871G>A	NP_776533.1:A291T	Present work
e^f^	—	NM_174108.2:c.890T>C	NP_776533.1:I297T	Graphodatskaya (2002)

Comparison with previously reported variants for the *MC1R* gene revealed that the SNP c.274G>A (NM_174108.2:c.274G>A), significantly associated with the black coat color in the Nelore breed in this study, is a novel variant. We propose designating the mutant allele “A” as *E* ^*Z1*^, where “E” follows the standard notation for *MC1R* (Extension) alleles, “Z” refers to its occurrence in zebu cattle, and the number 1 indicates its physical position preceding the next named variant in the gene. Additionally, the variant c.871G>A (NM_174108.2:c.871G>A), associated in the present study with the Dark skin phenotypic group (characterized by a typically darkened coat color phenotype), had been previously reported in databases, but without an established formal nomenclature. Therefore, we propose the designation of the mutant allele “A” as *E* ^*Z2*^, following the same nomenclature logic and using the number 2 because it is physically located downstream of the *E* ^*Z1*^ variant in the gene sequence.

Another important polymorphism found in the present work (Table 3) and also previously reported is the indel c.310 (NM_174108.2:c.310del) (Table 6), which eliminates reading‐frame continuity and produces a truncated 154‐aa protein instead of the full 317‐aa protein. Thus, in individuals homozygous for the deletion, genotyping beyond c.460 is biologically unnecessary (after stop codon), although extended genotyping was conducted in a subset to characterize downstream allele segregation and compute LD and haplotype reconstruction. This mutation is present in the H5 and H6 haplotypes (Table 4).

**FIGURE 1:**
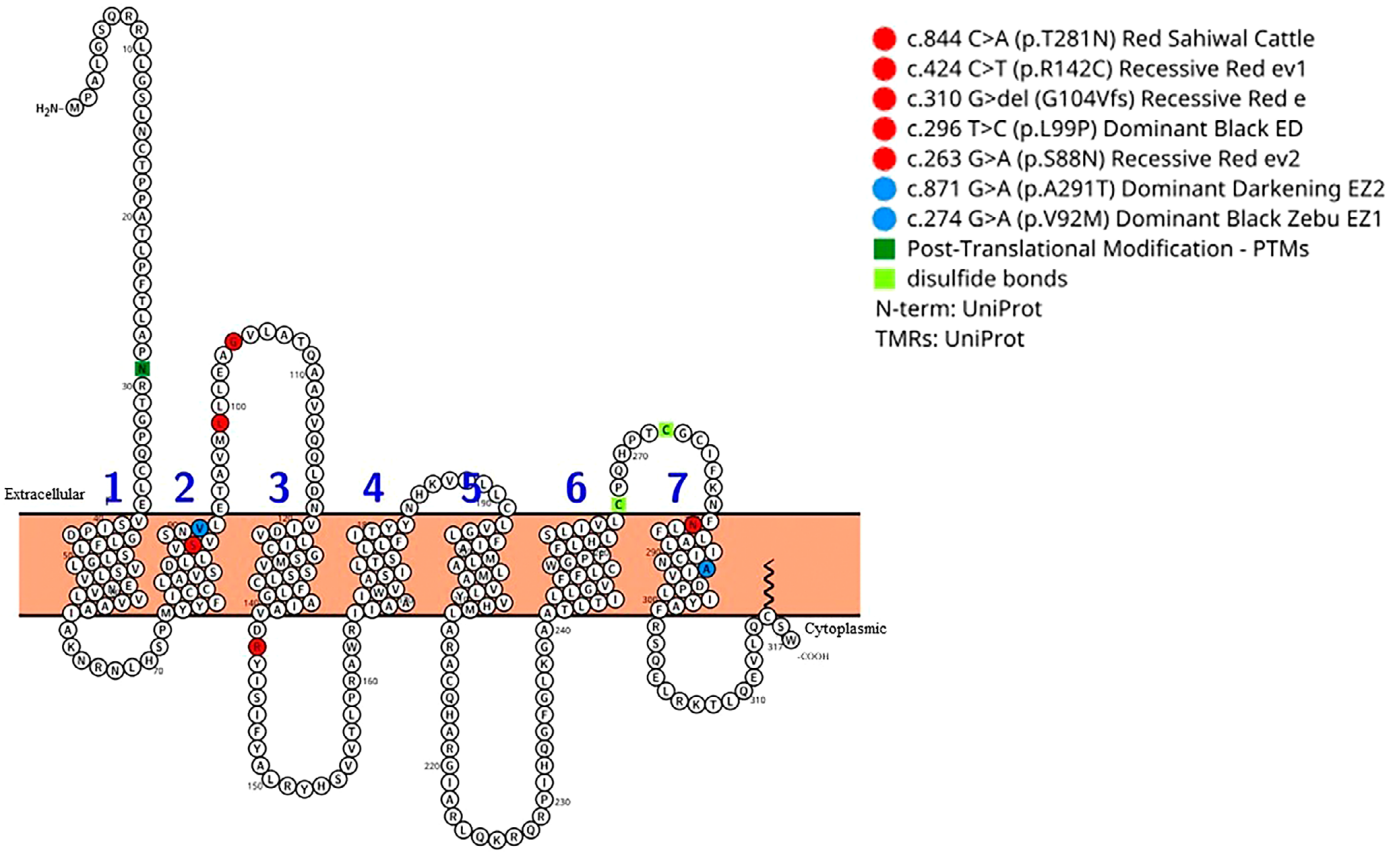
Schematic representation of the 2D topology of the bovine *MC1R* receptor, highlighting the seven transmembrane domains (TM1–TM7), the extracellular N‐terminus, and the cytoplasmic C‐tail. Some classic variants with proven effects on coat color are shown in red, and the novel variants identified in this study are shown in blue (E^Z1^ and E^Z2^). Additional markers indicate posttranslational modification (PTM) sites and disulfide bonds. Information is detailed in Table 6.

Additionally, a schematic representation of the protein encoded by the *MC1R* gene was generated (Figure 1), combining some variants with a known effect on bovine coat color (Table 6). The GPCR is composed of 317 amino acids and features seven transmembrane domains (TM1–TM7), an extracellular N‐terminus, and a cytoplasmic C‐terminal tail.

The classical variants previously reported in the literature with proven effects on coat color are highlighted in red, and the novel variants reported in the present study are in blue (Figure 1). The variants *E* ^*D*^ and *E* ^+^, as well as the recessive *e* ^*V2*^ and *e* are located in the first extracellular domain and the second transmembrane domain (TM2), affecting the binding regions of the receptor. Notably, the novel missense polymorphism *E* ^*Z1*^ is located in the vicinity of these same major‐effect variants, being anchored in TM2. This suggests that the association obtained through the analyses of the present study is biologically compatible with the previously reported classical variants.

In turn, the *E* ^*Z2*^ variant is located in the terminal region of the receptor, inserted into the seventh transmembrane region (TM7) near the cytoplasmic C‐terminal tail, potentially directly affecting the final communication of the GPCR with the intracellular environment.

The strategic positioning of these *E* ^*Z1*^ and *E* ^*Z2*^ mutations within the transmembrane domains supports the idea that both act through a gain‐of‐function mechanism, triggering the biosynthesis of eumelanin (dark pigment).

### 3.1 Gir Cattle

Significant associations were detected in scenarios contrasting solid colors and different white spotting patterns: GS2 (solid vs. spotted) (c.310 and c.583), GS6 (solid vs. choker) (c.583), GS7 (solid vs. less spots) (c.583), and GS10 (solid vs. more spots) (c.416, c.460, and c.583). In these cases, the SNP c.583C>T (NM_174108.2:c.583C>T) displayed the strongest association signal, which could initially suggest it as a causal mutation for the white spotting phenotype (Tables 2 and 5).

However, considering that the *MC1R* locus is not the primary biological responsible for white markings, and integrating these results with the haplotype and diplotype frequencies (Appendix S3), a new scenario, GS11 (red vs. spotted), was developed. In this scenario, solid yellow (Y) animals were excluded from the group due to their high genomic and diplotypic variability. As a result, the significance values for SNP c.583 and other SNPs disappeared, confirming that the previously observed association was not linked to the occurrence of white markings, but rather to the genetic signature of the solid yellow coat color (Appendix S5). This suggests that the yellow phenotype may be under epistatic effects or complex genetic interactions that are only expressed in specific genetic combinations.

Additionally, it was observed that the H4 haplotype and the H4/H4 diplotype are significantly more frequent in the red coat color group than in the other groups (yellow and dark skin) (Appendix S3). This pattern strongly suggests that the red coat represents the ancestral pattern of the Gir breed. Other color variations likely emerged later through spontaneous mutations accumulated on this “red” background (H4), being subsequently selected and fixed based on breeders' interests. This hypothesis would explain the greater genetic variability found in the less common phenotypes (yellow and dark skin) compared to the stability observed in the red coat.

Significant associations were also found for dark skin phenotypes: GS4 (dark skin vs. non‐dark skin) (c.274, c.310, c.583, c.663, and c.871) and GS9 (dark skin vs. solid coat) (c.416, c.460, c.583, c.663, and c.871). The most significant SNP in both scenarios was c.871G>A (*E* ^*Z2*^), which appears to be the likely causal mutation (Table 5). In dark‐skin individuals, the A allele showed higher frequency, as did heterozygous GA genotypes. Interestingly, no AA genotypes were detected in the entire Gir population sampled (Appendix S2).

The remaining associated polymorphisms segregate independently (*r* ^2^ < 0.8), indicating that additional additive effects may also be present (Appendix S4). The variants c.274 (*E* ^*Z1*^) and c.310 exhibit minor allele frequencies near or below 0.05 in the group (data not shown), suggesting that expanded sampling will be necessary to confirm the robustness of these associations.

### 3.2 Nelore Cattle

For Nelore cattle, in scenario NS2 (black vs. non‐black) which tested for association with black coat phenotype, SNPs c.274 (E^Z1^), c.583, c.663 (NM_174108.2:c.663T>C) and c.871 were significant (Tables 2 and 5). Among these, c.274G>A showed the strongest association and is suggested to be the causal mutation. This variant likely acts in a dominant manner, as the presence of a single “A” allele is associated with the black phenotype. The frequency of the A allele was 0.46 among black animals but only 0.01 in non‐black individuals (data not shown). SNPs c.583 and c.663 segregated independently from c.274 (*r* ^2^ < 0.8), suggesting possible additive contributions (Appendix S4), whereas c.871 exhibited complete linkage to c.274 in this specific population.

The analysis of haplotype and diplotype frequencies reinforces the identification of this variant. It is observed that the “Indicine consensus haplotype” pattern (H4 haplotype and H4/H4 diplotype) predominates in non‐black animals. However, this pattern is disrupted in black animals, which primarily present the H9 haplotype (carrier of the E^Z1^ mutation) and a very high frequency of the H4/H9 heterozygous diplotype (Appendix S3). This strongly suggests that the black coat in Nelore originated from a point mutation (H9) that altered the ancestral genotype (H4), causing phenotype modification and subsequent selection.

Regarding phenotype classification in Nelore cattle, one white‐coated individual (classified as non‐black) (Appendix S1–S1.3.1) carried the genotypes c.274 G/A, c.583 T/C, c.663 C/T and c.871 G/A. This animal presented a fully white coat yet retained black pigmentation inside the ears, contrasting with the typical pink ear interior of the breed (Appendix S1–S1.3.1). We hypothesize that this individual is genetically black (*E* ^*Z1*^ carrier), but its body coat color is masked by extreme white spotting (epistasis), which is consistent with its origin from a Spotted Nelore herd.

Another observation is that within the black group, two of the 13 animals displayed genotypes that differed from the majority. These animals exhibited reddish tones on the ventral region and other body areas (Appendix S1–S1.3.2), suggesting that the interaction with other pigmentation genes may also be influencing the final intensity of the coat darkening.

In scenario NS5 (Blue vs. Not Blue) (Table 2), SNP c.296 (*E* ^*D*^) was highly significant, with the C allele being the likely causal variant for the blue coat phenotype (Table 5). However, sample size was extremely limited (*n* = 3), as this phenotype is extremely rare in Zebu breeds, so more individuals must be included in future analyses to confirm the association. Scenarios NS3 (Red vs. Not Red) and NS6 (Spotted vs. Solid) (Table 2) also revealed the same significant SNPs, but based on allele distribution, it is likely that these results are confounded by the presence of black coat variation, preventing a clear interpretation of the true SNP effect on these phenotypes.

### 3.3 Depigmentation

Across all scenarios comparing pigmented versus depigmented animals (Table 2), including Guzerá (GUS1), Gir (GS3), Sindi (SS1), and the combined analysis of all breeds (All1), the c.310del mutation showed strong association, with the exception of Nelore cattle (NS7) (Table 5; Appendix S5). All depigmented individuals from Guzerá, Gir, and Sindi carried at least one copy of the deletion (Appendix S3). In Guzerá specifically, this variant was also associated with the roan coat pattern (GUS1 and GUS2) (Tables 2 and 5).

These findings indicate that the c.310del predisposes individuals to depigmentation, as every depigmented animal carried the allele. However, incomplete penetrance was observed, since some deletion carriers remained normally pigmented (Appendix S3). This suggests that the depigmentation phenotype is likely modulated by epistatic interactions, possibly involving yet unidentified genetic modifiers.

## 4 Discussion

The main *MC1R* principal variants associated with coat phenotype in indicine cattle were: c.274G>A (*E* ^*Z1*^) (NM_174108.2:c.274G>A), associated with black coat color in Nelore cattle; c.871G>A (*E* ^*Z2*^) (NM_174108.2:c.871G>A), which contributes to darkening coat color in dark skin coats variation in Gir cattle; the c.310del (NM_174108.2:c.310del), which confers susceptibility to depigmentation in Sindi, Guzerá, and Gir cattle breeds; the c.583C>T (NM_174108.2:c.583C>T) appears to be associated with the occurrence of solid yellow coat color and also with a complex gene interaction effect.

The c.274G>A SNP (NM_174108.2:c.274G>A) is reported here for the first time, representing a missense mutation (p.Val92Met) in which the A allele is associated with black coat color in Nelore and may therefore serve as a molecular marker. In taurine breeds, the G allele is fixed. It is worth noting that a lower frequency of black phenotype is observed in indicine cattle, with registration being recently permitted in Nelore, Gir, and Brahman breeds (ABCZ 2025).

The amino acid NP_776533.1:Val92Met, originating from the c.274G>A polymorphism (Table 3; Figure 1), is located in the second transmembrane domain (TM2). This position is physically adjacent to residue 88, where the p.Gly88Ser substitution has been associated with the recessive red *e* ^*V2*^ phenotype (Hauser et al. 2022). Although in silico analysis classified the p.Val92Met variant as tolerated (SIFT = 0.13), its location in a conserved alpha helix of TM2 suggests a relevant structural role. Both genetic variants lead to amino acid substitutions in a highly conserved region of TM2, close to the first extracellular domain, which harbors the most studied regulatory sites of the *MC1R* gene (*E* ^*D*^, E^+^, and *e*) (Klungland et al. 1995; Rouzaud et al. 2000; Santana et al. 2021; Hauser et al. 2022; He et al. 2022).

While the allele e^v2^ (Hauser et al. 2022) appears to stabilize the inactive conformation (red), we suggest that the substitution of valine for methionine at position 92 exerts a gain‐of‐function effect, keeping the receptor constitutively active. The strong genotypic and haplotypic segregation (Appendices S2 and S3) in Nelore cattle between black and non‐black animals reinforces that, despite the conservative prediction of SIFT, this mutation is the main determinant of black coat color in this breed.

The structural integrity of the TM2 helix is crucial for the stability and activation kinetics of the G protein‐coupled receptor (GPCR). Hauser et al. (2022) proposed that the introduction of a polar residue (Serine) at position 88 could form additional hydrogen bonds, stabilizing the inactive conformation of the receptor and resulting in red coat color. In contrast, our results associate the substitution of Valine for Methionine (an amino acid with a larger side volume) at position 92 with the dominant black coat color in the Nelore breed. We suggest that this alteration in TM2 exerts an opposite effect, possibly stabilizing the active conformation of the GPCR or increasing its constitutive activity. This gain‐of‐function mechanism would promote the continuous synthesis of eumelanin, independent of hormonal stimulation, justifying the occurrence of the black coat color observed in carriers of the *E* ^*Z1*^ allele.

The c.871G>A SNP (NM_174108.2:c.871G>A) was associated with dark phenotypes in Gir cattle. Maciel et al. (2024) identified an association between a region on BTA18, proximal to *MC1R*, with the dark skin phenotype in Gir cattle, consistent with that observed in the present study. The c.871G>A SNP has been previously reported in bovine populations (Zhang et al. 2014) and in other species of the genus Bos (Chen et al. 2009; Xi, Liu, et al. 2012; Xi, Wu, et al. 2012), although without a clear association with coat color differentiation. However, in silico analyses indicate that this missense mutation alters the protein structure with a potentially deleterious functional effect (SIFT close to 0), as observed in our results (Table 3).

Furthermore, the haplotype reconstructions by Matsumoto et al. (2020) were not phased, as this step is not mentioned in their materials and methods, which may have led to hasty inferences about the effect of the genotype. The interpretation of these authors seems not to consider the functional biology of the variants present. The high frequency of the recessive allele *e* (c.310del) in that population implies the occurrence of a premature stop codon at position 104, which truncates the protein before position c.871. Biologically, it is unlikely that a variant located downstream of an early termination signal would influence the phenotype, since the C‐terminal region of the protein is not even translated in carriers of the e allele.

Therefore, the evidence presented in this study is more compelling, associating the c.871G>A (NM_174108.2:c.871G>A) variant with black coat and dark skin in Gir cattle, and shows greater biological compatibility with the role of *MC1R* in eumelanin synthesis.

The variant c.871G>A (p.Arg291His) (NM_174108.2:c.871G>A), identified as the *E* ^*Z2*^ allele, is located in the seventh transmembrane domain (TM7). The work of Goud et al. (2021) highlights that the transmembrane helices of *MC1R* in zebu cattle are regions of high evolutionary conservation, suggesting that mutations in these locations tend to impact intracellular signaling. Our results corroborate this premise, since the in silico analysis indicated a deleterious functional effect for this substitution (SIFT = 0.03, Table 3). The exchange of an Arginine (basic) for a Histidine at position 291 alters the electrostatic properties of the TM7 helix, which may prolong the active state of the receptor and favor the continuous synthesis of eumelanin, justifying the association with dark skin in Gir cattle and black coat in Nelore cattle.

The associations detected for c.871 (NM_174108.2:c.871G>A) may contribute to explaining coat color determination in Gir cattle and could serve as molecular markers for selection. However, pigmentation in this breed is known to be polygenic and influenced by epistatic interactions (Maciel et al. 2024). The findings presented here provide an initial step toward a more detailed understanding of the genetic architecture underlying coat color expression in Gir cattle.

Although c.583C>T has been previously reported, no studies had linked it to coat color variation in cattle (Hanna et al. 2014; Goud et al. 2019; Jiang et al. 2021). It can be understood that this variant does not cause a sudden change in the color phenotype but rather acts through gene interactions to determine the final phenotype.

Another widely discussed mutation affecting coat and skin pigmentation in cattle occurs at c.310del (NM_174108.2:c.310del), which induces a frameshift and premature stop codon. This variant has been associated with red coat coloration in taurine cattle (Klungland et al. 1995; Matsumoto et al. 2020) and with depigmentation in indicine breeds (Santana et al. 2021).

Bovo et al. (2021) also reported that *MC1R* mutations may contribute to muzzle pigmentation variation (Pink, gray, or Black) in Reggiana cattle. Santana et al. (2021) reported that homozygosity for the c.310del results in depigmentation in Guzerá cattle. The present study further demonstrates that this deletion is also associated with the roan phenotype in Guzerá (Appendix S1–S1.3.3). Hauser et al. (2022) indicate that animals that have at least one *e* ^*V1*^ allele appear to have a darker snout than animals that have the *e* and *e* ^*V2*^ alleles. Additionally, in the photos presented by Han et al. (2011) exemplifying the genotypes of the *MC1R* gene and the *ASIP* gene in cattle of Korean origin, it can be observed that animals with the e/e genotype have a depigmented snout, while animals with at least one variant of the *E* ^+^ allele have a snout with greater pigmentation, except in the *E* ^+^ */e A* ^*BR*^ */A* ^*BR*^ genotype, which indicates the occurrence of gene interactions for the final determination of the snout.

Our results indicate that the variant does not act in a strictly deterministic manner, but rather exhibits incomplete penetrance, whereby the deletion increases the likelihood of depigmentation but expression appears to depend on an additional, possibly epistatic, genetic factor yet to be identified, according to what was discussed by Hauser et al. (2022). We also observed variable expressivity, as the same deletion can result in two distinct phenotypes–roan and depigmented–likely driven by this same epistatic interaction.

The deletion also predisposes individuals to depigmentation in Sindi and Gir, but not in Nelore. This is consistent with findings reported by Campos et al. (2025), who detected no evidence implicating *MC1R* in depigmentation mechanisms in Nelore cattle, but rather the *KIT*, *MITF*, and *EDNRB* genes. Together, these results suggest that depigmentation in indicine cattle represents a case of genetic heterogeneity, with different causal variants across breeds.

The c.296C>T SNP has been extensively studied in taurine cattle, where the C allele is designated *E* ^*D*^ and the T allele *E* ^+^, corresponding respectively to black and wild coat phenotypes (Klungland et al. 1995; Matsumoto et al. 2020; Hauser et al. 2022). In the present study, this SNP was suggested as a possible determinant of the blue coat phenotype in Nelore cattle (Appendix S1–S1.2.3g). Two of the three blue‐coated animals carried the *E* ^*D*^ */E* ^+^ genotype, whereas all other 294 animals from all remaining phenotypes exhibited *E* ^+^ */E* ^+^, demonstrating that *E* ^*D*^ is rare in indicine cattle. This likely reflects taurine introgression, which may have contributed to the origin of the blue phenotype, consistent with the findings of Luo et al. (2025).

Haplotype H4, characterized by the c.296T (E^+^) allele and the c.583C>T and c.663T>C SNPs, was identified across all breeds in the present study, which is in agreement with literature data for other breeds of indicine origin and constitutes the previously described TTC haplotype (Hanna et al. 2014; Goud et al. 2019, 2021; Jiang et al. 2021) thus, the term “Indicine consensus haplotype” is suggested for this formation. The characterization of the zebuine *MC1R* gene reveals a structural divergence from the taurine pattern, highlighting the c.583C>T polymorphism as a phylogenetically conserved marker in Asian and native Indian breeds and an essential component of the basal indicine identity (Goud et al. 2019, 2021; Jiang et al. 2021). Furthermore, evidence suggests that the C allele may be associated with dark coloration naturally uncommon in Zebu cattle which explains the high frequency of the T allele in these animals (Goud et al. 2019, 2021; Jiang et al. 2021; Luo et al. 2025; Genuíno et al. 2025).

Consistent with this, H4 was observed at a higher frequency in white and red coated animals, phenotypes known to favor tropical adaptability and the easy fixation of modern breed qualification, with this genetic background resulting from a remote evolutionary history (Genuíno et al. 2025; Luo et al. 2025). Additionally, the darkening variants *E* ^*Z1*^ (c.274G>A) and *E* ^*Z2*^ (c.871G>A) occur upon this stable H4 genomic architecture, as evidenced by the high linkage disequilibrium (LD) with positions 583 and 663. This demonstrates that pigment modulation toward dark coloration in Zebu cattle depends on mutations that interact with the conserved H4 structure, requiring regulatory complexity for phenotypic modification (Hanna et al. 2014).

*ASIP* alleles have been reported in zebu cattle breeds (Trigo et al. 2021, 2023) and are associated with regional darkening of the coat in Nelore cattle and Brahman cattle. To further elucidate the genetic architecture of coat color in indicine cattle, a comprehensive characterization of these alleles across breeds, together with an evaluation of their epistatic interactions with the *MC1R* polymorphisms reported herein, is warranted.

The investigation of *MC1R* variation in zebu breeds enabled the identification of molecular markers with potential application in marker‐assisted selection, particularly for avoidance of depigmentation phenotypes, a disqualifying phenotype for animal registration in breeder associations across all breeds.

## 5 Conclusion

In conclusion, this study expands current knowledge on the influence of *MC1R* in indicine cattle breeds and identifies molecular markers with potential use in selection strategies. Coat color in indicine cattle is a complex trait influenced by multiple genes and interactive effects, and the present findings contribute to advancing the understanding of its genetic architecture. By clarifying the role of *MC1R* in pigmentation, this work helps bridge an existing gap regarding coat color determination in indicine cattle.

## Author Contributions

**Silel V. S. A. Maciel:** writing – original draft, methodology, validation, formal analysis, writing – review and editing, investigation. **Ingrid P. P. Oliveira:** formal analysis, methodology, writing – review and editing. **Beatriz B. Senes:** methodology, formal analysis, writing – review and editing. **Carolaine J. S. Santana:** methodology, formal analysis, writing – review and editing. **Jackeline S. Alves:** methodology, formal analysis. **Edilane A. da Silva:** data curation, writing – review and editing. **Fernando O. Franco:** writing – review and editing, data curation. **Bárbara C. F. da Silva:** data curation, writing – review and editing. **Fernando C. Cairo:** data curation, writing – review and editing. **Frederico C. Cairo:** data curation. **Aníbal E. Vercesi‐Filho:** data curation, writing – review and editing. **Raphael B. Costa:** funding acquisition, writing – review and editing, formal analysis. **Gregório M. F. de Camargo:** conceptualization, writing – review and editing, supervision.

## Funding

The authors wish to thank the “Coordenação de Aperfeiçoamento de Pessoal de Nível Superior (Capes)” for the fellowship of the first author, the “Pró‐Reitoria de Extensão da Universidade Federal da Bahia (PROEXT—UFBA)”, Finep (1334/13), INCT‐CA (408487/2024‐0), and Fapemig for funding.

## Disclosure

Permission to reproduce material from other sources: The authors allow the reproduction of this material.

## Ethics Statement

This study was approved by the Animal Ethics Committee of the Escola de Medicina Veterinária e Zootecnia of the Universidade Federal da Bahia (project number approval 29/2021).

## Conflicts of Interest

The authors declare no conflicts of interest.

## Supporting information


**Appendix S1:** Names, coat colors, and photos of indicine cattle breeds studied.

**Appendix S2:** Frequency of the polymorphisms in *MC1R* gene in zebu cattle.

**Appendix S3:** Frequency of the haplotypes and diplotypes in the *MC1R* gene in zebu cattle.

**Appendix S4:** Linkage disequilibrium parameters of SNPs in the *MC1R* gene in zebu cattle.

**Appendix S5:** Kruskal–Wallis test for SNPs of the *MC1R* gene.

**Appendix S6:** Reported variants of *M* *C1R*.

## Data Availability

The sequence data were deposited in GenBank and can be accessed under the following numbers: PQ461102 (Gir), PQ461103 (Sindi), PQ461104 (Guzerá), PQ461105 (Tabapuã), PQ461106 (Brahman), PQ461107 (Nelore), and PQ461108 (Indubrasil).
