# Supplementary material for: Influence of MC1R Gene Variants on Coat Color of Indicine Cattle Breeds
Source: Anim Genet. 2026 May 2;57:e70113. doi: 10.1002/age.70113 (PMC13135194; doi:10.1002/age.70113)
Supplement: Supplementary file 1 — Appendix S1: Names, coat colors, and photos of indicine cattle breeds studied. [file AGE-57-0-s006.docx]

**Supplementary Material 1**

1. **Names, coat colors and photos of indicine cattle breeds studied.**
   1. **Names of indicine breeds^*^**

| **Portuguese** | **English** |
| --- | --- |
| Gir | Gyr |
| Nelore | Nellore |
| Sindi | Sindhi |
| Tabapuã | Tabapua |
| Indubrasil | Indubrasil |
| Guzerá | Guzerat |

^*^Following recommendation of DAD-IS/FAO, we used breed names in Portuguese (official country language where animals are raised).

- 1. **Examples of coat colors in zebu cattle registered in the Brazilian Zebu Cattle Breeder Association (ABCZ, in Portuguese acronym)**
     1. **Coat color Gir cattle**


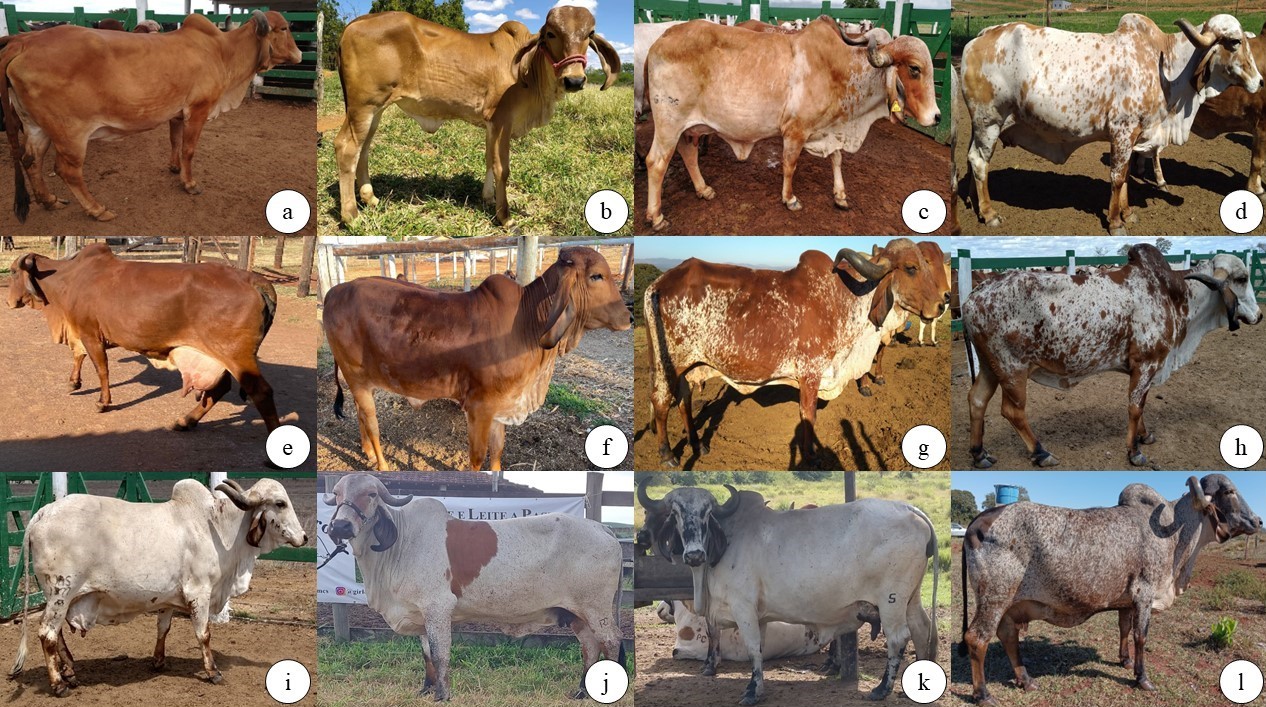


**Legend coat colors Portuguese (English): a:** Amarela (Yellow); **b:** Amarela Gargantilha (Yellow Choker)^1^; **c:** Amarela Chitada (Yellow Calico)^2^; **d:** Chitada de Amarela (Calico Yellow)^3^; **e:** Vermelha (Red); **f:** Vermelha Gargantilha (Red Choker)^1^; **g:** Vermelha Chitada (Red Calico)^2^; **h:** Chitada de Vermelha (Calico Red)^3^; **i:** Chita Clara (Calico Clear)^4^; **j:** Moura de Vermelha (Dark Skin, Red Head)^5^; **k:** Moura Clara (Dark Skin, Light Coat Color)^6^; **l:** Moura Escura (Dark Skin, Dark Coat Color)^7^. **Photos Authors: a**, **c**, **h** and **i** by Fernando Cairo; **b** by José Paulino; **d** by Moacir Dias; **e** and **f** by Edilane Silva; **j** by Matheus Silva; **k** by Aníbal Vercesi; **l** by Jacinto Filho.

^1^ Amarela/Vermelha Gargantilha: spots on the dewlap.

^2^ Amarela/Vermelha Chitada: few spots on the body.

^3^ Chitada de Amarelo/Vermelho: many spots on the body.

^4^ Chita Clara: white.

^5^ Moura de Vermelho or Rosilha: white with fully or partially red ears and head.

^6^ Moura Clara: white with fully or partially black ears and head.

^7^ Moura Escura: white, with a predominance of dark color and a black head and ears.

- - 1. **Coat color Nelore cattle**


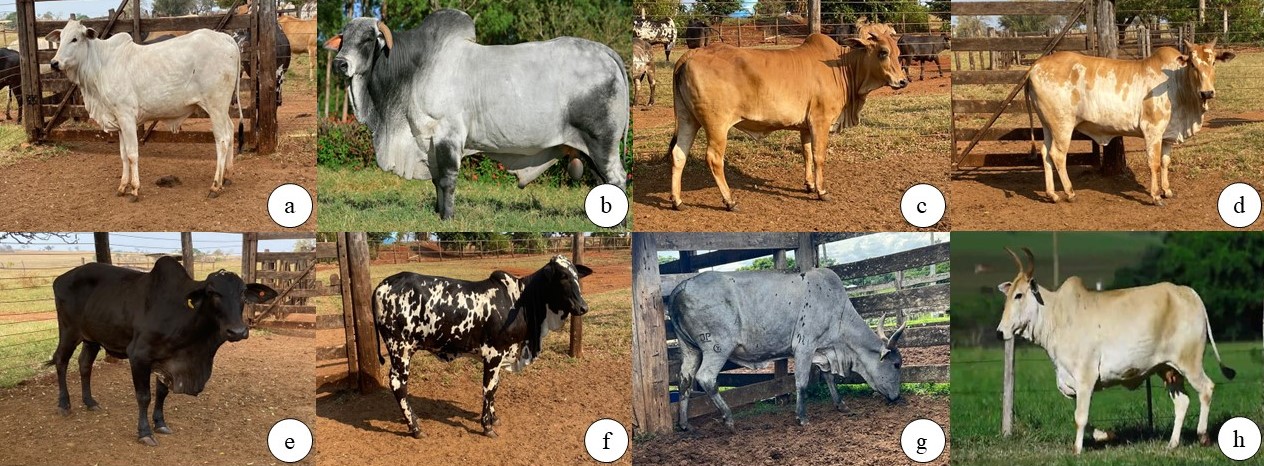
**Legend coat colors Portuguese (English):** **a:** Branca (White); **b:** Cinza (Grey); **c:** Vermelha (Red); **d:** Pintada de Vermelha (Spotted Red); **e:** Preta (Black); **f:** Pintada de Preta (Spotted Black); **g:** Baeta (Blue)^1^; **h:** Amarela (Yellow). **Photos Authors:** **a**, **c**, **d**, **e** and **f** by Eduardo Agreli; **b** by 3A Genética; **g** by Rosa Carvalho; **h** by Manoel Falcão.

^1^ Baeta: dark grey with bluish tones and intense black color in the mucous membranes and areas with few hairs.

- - 1. **Coat color Sindi cattle**


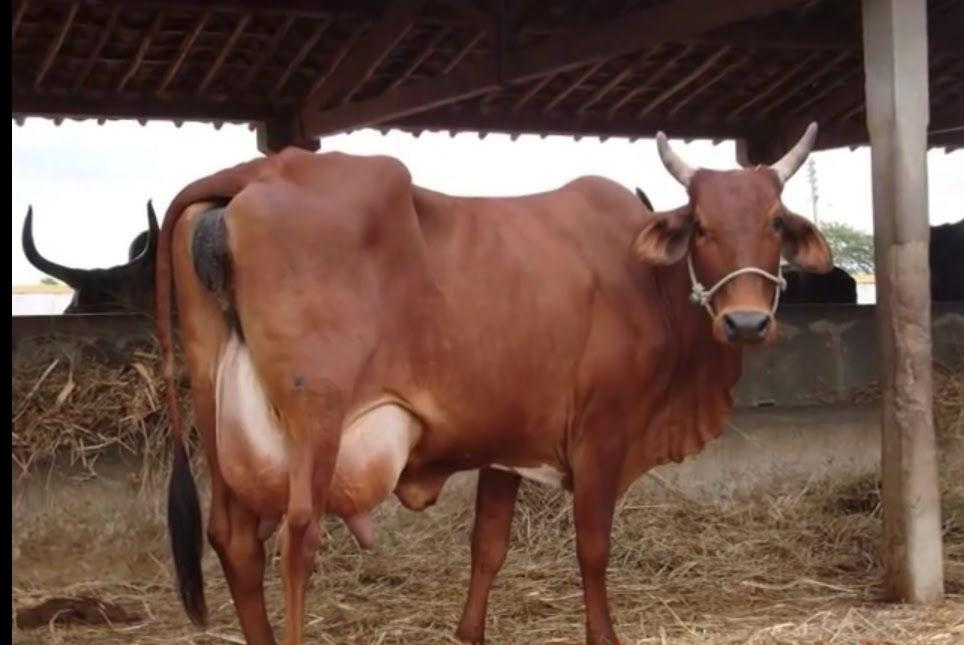


**Legend coat colors Portuguese (English):** Vermelha (Red);

**Photo Author:** by Daniel Dantas.

- - 1. **Coat color Indubrasil cattle**


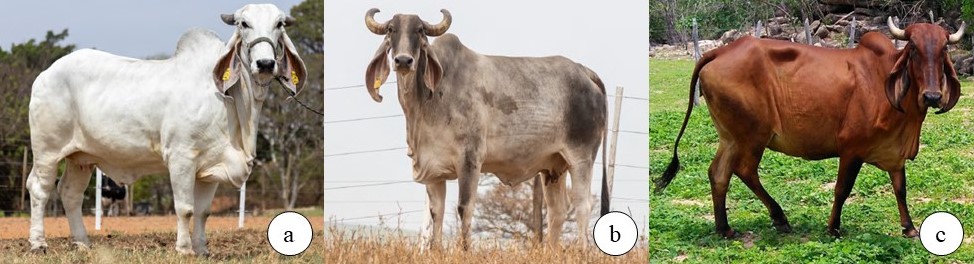


**Legend coat colors Portuguese (English):** **a:** Branca (White); **b:** Cinza (Grey); **c:** Vermelha (Red).

**Photos Authors:** **a** and **b** by Fazenda Iracema Indubrasil; **c:** Indubrasil do General.

- - 1. **Coat color Tabapuã cattle**


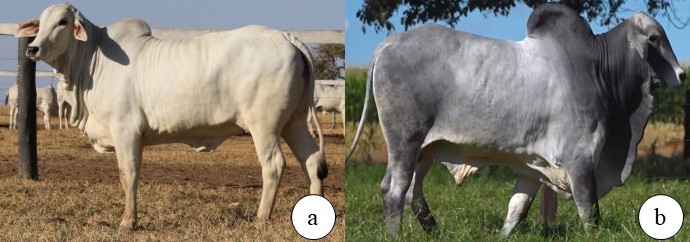


**Legend coat colors Portuguese (English):** **a:** Branca (White); **b:** Cinza (Grey).

**Photos Authors:** **a** by Associação Brasileira de Criadores de Tabapuã; **b:** by Tabapuã da Gê 05.

- - 1. **Coat color Guzerá cattle**


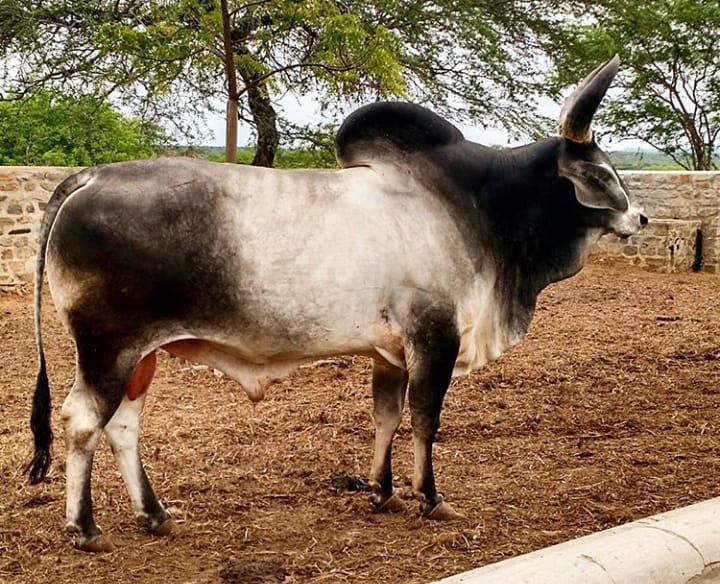


**Legend coat colors Portuguese (English):** Cinza (Grey).

**Photo Author:** by Daniel Dantas.

- - 1. **Coat color Brahman cattle**


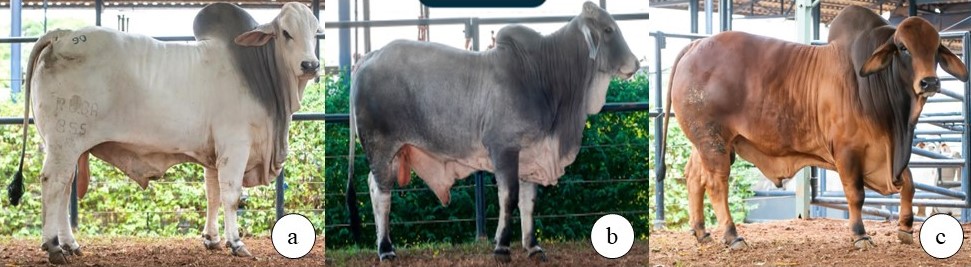


**Legend coat colors Portuguese (English):** **a:** Branca (White); **b:** Cinza (Grey); **c:** Vermelha (Red).

**Photos Authors:** **a, b** and **c** by Associação dos Criadores de Brahman do Brasil.

- 1. **Animals registered in ABCZ, but with peculiarities in their coat color**

**1.3.1. Nelore with black ears**


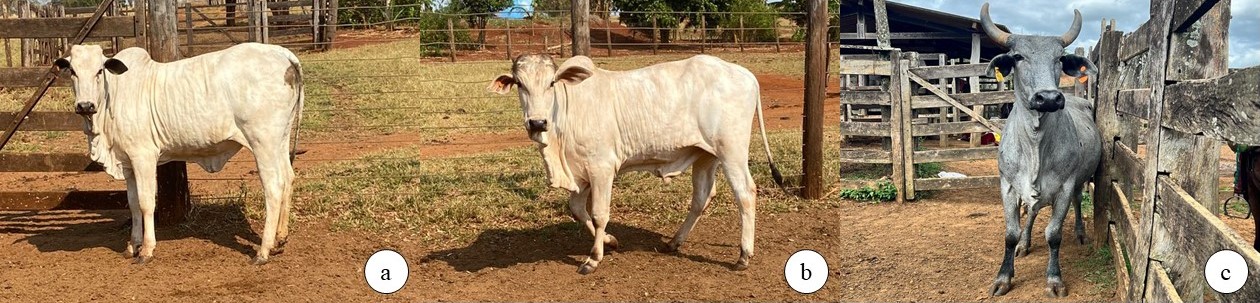


**Legend: a:** Animal with white coat color with black skin inside the ears (uncommon); **b:** Animal with white coat color with pinkish skin inside the ears (most common); **c:** Animal with blue coat color and on the inside of the ears the skin color is black.

**Photos Authors:** **a** and **b** by Eduardo Agreli; **c:** Rosa Carvalho.

- - 1. **Black Nelore**


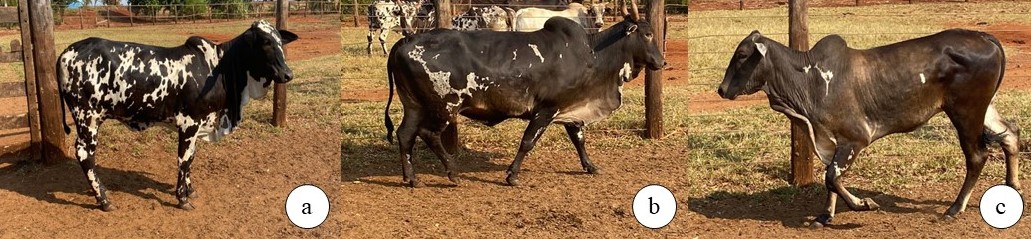


**Legend: a:** Animal with standard Black Spotted coat color; **b-c:** Animals apparently with Black Spotted coat color, but with reddish color in ventral region (b) and other body parts (c).

**Photos Authors:** **a**, **b** and **c** by Eduardo Agreli.

- - 1. **Guzerá with reddish hair**

**
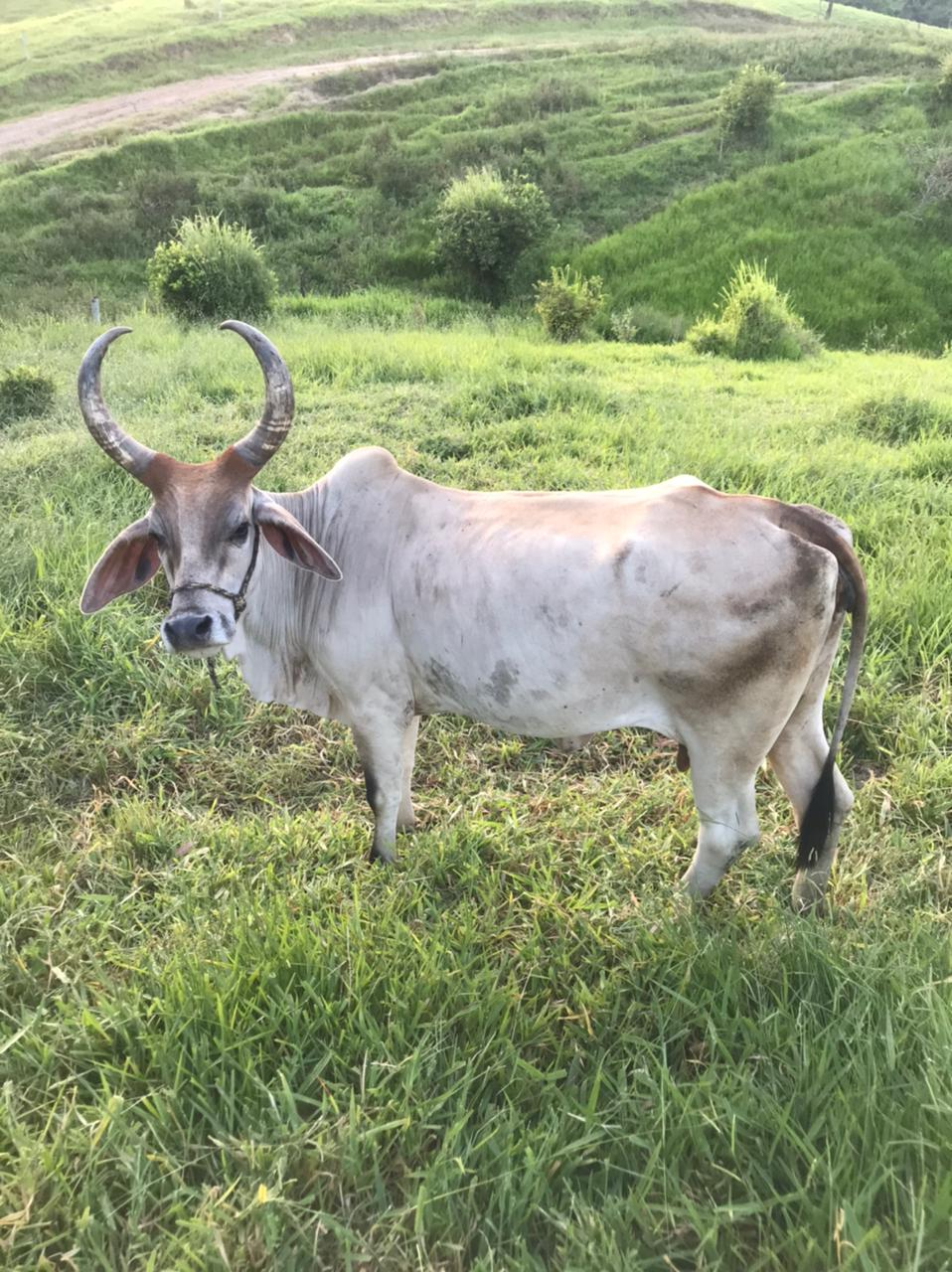
**

**Legend coat colors Portuguese (English):** Debruada^1^ (Roan)

^1^Debruada: grey coat color with some body regions with reddish hairs

**Photo Author:** Davi Teixeira.

- 1. **Animals unregistered in ABCZ due to mucosa depigmentation - Pigmented animal (left) x depigmented animal (right)**


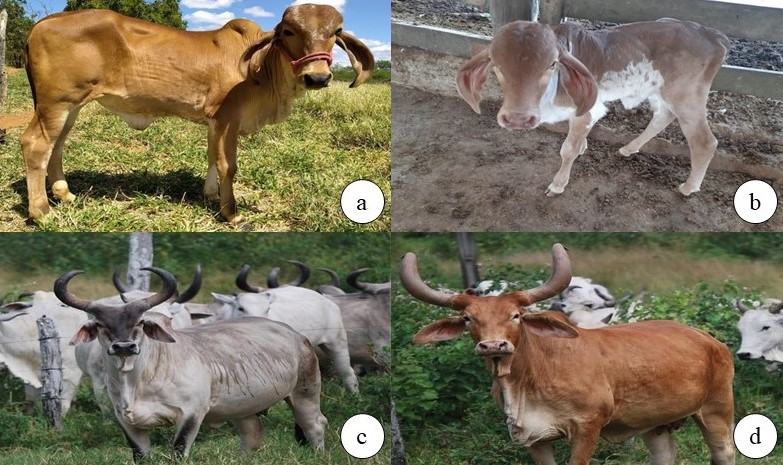


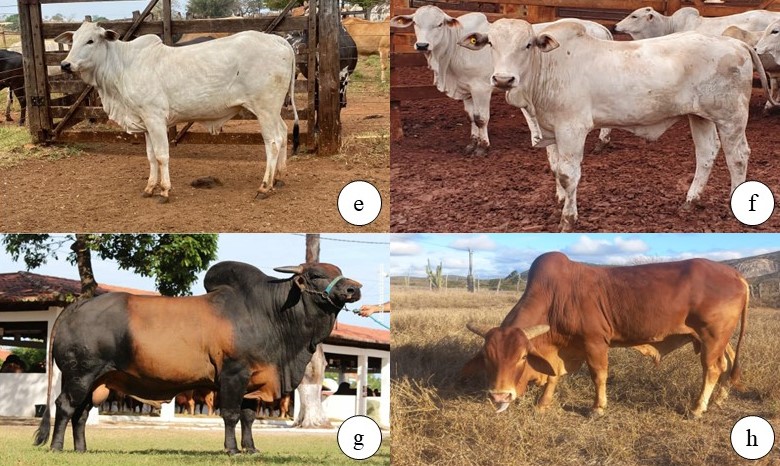


**Legend: a** and **b:** Gir cattle; **c** and **b:** Guzerá cattle; **e** and **f:** Nelore cattle; **g** and **h:** Sindi cattle.

**Photos Authors:** **a:** José Paulino; **b:** José Costa**; c** and **d:** Davi Teixeira; **e:** Eduardo Agreli; **f:** Júlia Valente**; g:** Daniel Dantas; **h:** Cézar Mastrolorenzo
